# Editorial: Integrated therapy approaches in schizophrenia: Evidence and limitations

**DOI:** 10.3389/fpsyt.2023.1142493

**Published:** 2023-02-01

**Authors:** Daniel R. Mueller

**Affiliations:** University Hospital of Psychiatry and Psychotherapy, University of Bern, Bern, Switzerland

**Keywords:** schizophrenia, integrated therapy, psychotherapy, treatment adherence, cognitive remediation

## The definition of integrated therapy

Do Integrated Therapy Approaches (ITA) really represent a promising treatment possibility for schizophrenia patients? Before discussing this question from a clinical and scientific perspective, its objective should be defined first. Various recently published national psychiatric guidelines on psychosocial interventions are based on empirical evidence regarding the efficacy of each approach. In common they have the following recommendations of the highest degree for psychotherapy approaches, even if in some counties psychotherapy does not reach the absolute highest degree of recommendation: Cognitive Behavior Therapy for psychosis (CBTp), Psychoeducation and Family therapy (PEFT), Social Skills Trainings (SST), and Cognitive Remediation (CR). It also has to be mentioned that Assertive Community Treatment and Supported Employment are given an equal status in North America. During the last decades, other therapy approaches have found broad acceptance and high recommendation as well. This includes for example Meta-Cognitive Training [MCT, (1)] and the so-called third wave of CBTp. Going back to the question posed at the beginning of this editorial, one of the very first definitions of ITA goes back to the Integrated Psychological Therapy (IPT) of Roder et al. (2), defining ITA as a combination of at least two of the described therapy approaches within an integrated, comprehensive therapy concept. ITA therefore is based on neurocognitive therapy goals used in CR, which is combined with intervention goals out of CBTp, SST, PEFT or others such as social cognition, meta-cognition, assertive community treatment and so on.

## What we already have

Following this definition, we then have a few well evaluated ITAs, which altogether have an impact on the efficacy of CR in general (3). Possibly the first of them is represented by the Integrated Psychological Therapy [IPT; (2), translation from German language] which integrates treatment aims focusing on neurocognitive and social cognitive functions (CR) together with social and communication skills (SST) in a group setting. Some meta-analyses support the efficacy of IPT (4). Further ITAs have been developed in the United States: Cognitive Enhancement Therapy [CET; (5)] integrates interventions on neuro- and social cognition and innovatively included PC-based exercises. Neurocognitive Enhancement Therapy (NET; (6)) and the thinking skills and work program (7) represent further developments of CET in adding supported employment. On the other hand, the Neuropsychological Educational to Rehabilitation [NEAR; (8)] combined NR with educational tools of PEFT. The world-wide distributed and successfully evaluated MCT (9) combines meta-cognition with NR and may be closer to CBTp than CR, even if containing exercise on neurocognition. Finally, Integrated Neurocognitive Therapy (INT) combines partly PC-based exercises to all from US National Institute of Mental Health defined neurocognitive and social cognitive functions with some PEFT and CBTp techniques (10). In this theme issue, we find a contribution to a further ITA not yet discussed: In a feasibility study with inpatients suffering of early psychosis, Hardenberg et al. evaluated a mindfulness-based approach focusing emotion regulation and wellbeing (“feel-good group”) combined with psychoeducation, self-monitoring techniques and other third-wave approaches including acceptance and commitment therapy, emotion-focused therapy, compassion-focused therapy, and schema therapy. Results suggest good acceptance by patients and some improvements in proximal outcome and symptoms.

## What we still need

All of these ITAs are strongly related to pharmacological interventions in schizophrenia patients. But only few studies have controlled the interaction of pharmacological and psychosocial interventions, even when these two intervention modalities can be defined as ITA itself. Consequently, in an article of this research theme Giordano et al. reviewed and discussed the available literature to detect possible factors of influence on treatment adherence for pharmacological agents. This is of importance for good reasons: (1) ongoing antipsychotic treatment prevent relapses, (2) psychosocial intervention may help to maintain treatment adherence, and (3) integrated models for schizophrenia support evidence for the impact of various cognitive, social and motivational functions representing treatment aims in ITA and have an impact on treatment adherence. The authors conclude that researchers and clinicians should examine these impact factors to improve outcome.

Every medal consists of two sides: the complexity of ITA represents the discussed benefit of ITA but it also represents its' disadvantage, as we do not know what really works. For example with IPT, which targets neuro- and social cognition and various social skills, it is unclear from what patients really benefit. We know that adding social cognitive interventions to the ones on neurocognition increases cognitive outcome (11, 12), but we do not know whether interventions on neurocognition are necessary at all. In the study design of a large RCT, Wölwer et al. compare the Integrated Social Cognitive and Behavioral Skill Therapy (ISST) with standard neurocognitive remediation. ISST comprises social cognitive domains that are tightly integrated to produce additional benefits in behavioral skills like conversation, assertiveness and vocational/work skills and vis-versa (Wölwer et al.). Therefore, the ISST-rationale corresponds strongly with the integrated model assumption that the whole is more than the addition of its parts. Results may give some evidence on its treatment aims regarding controls, too.

Another example of an even broader definition of ITA offers the study protocol of Chapellier et al.: here a classic ITA approach is combined with brain stimulation using repetitive transcranial magnetic stimulation (rTMS). Since the main focus in this study protocol concerned the treatment of gesture deficits in schizophrenia outpatients, continuous theta burst stimulation (cTBS) was used. On the other hand, general INT has been reduced to the social cognitive part with the addition of only verbal memory and working memory tasks out of the neurocognitive part. The comparison of this ITA with controls receiving sham INT (unspecific leisure time activities) or sham cTBS allows to detect what works. These two study protocols demonstrate a possible direction of future ITA research, where new approaches also consider medical factors of influence.

## Author contributions

The author confirms being the sole contributor of this work and has approved it for publication.
